# Leisure-Time Physical Activity and Cardiovascular Disease Risk Among Hypertensive Patients: A Longitudinal Cohort Study

**DOI:** 10.3389/fcvm.2021.644573

**Published:** 2021-05-28

**Authors:** Jiqing Li, Zhentang Zhang, Shucheng Si, Fuzhong Xue

**Affiliations:** ^1^Department of Biostatistics, School of Public Health, Cheeloo College of Medicine, Shandong University, Jinan, China; ^2^Healthcare Big Data Research Institute, Cheeloo College of Medicine, Shandong University, Jinan, China; ^3^Qingdao Huangdao District Center for Disease Control and Prevention, Qingdao, China

**Keywords:** leisure-time physical activity, cardiovascular disease, time-depended confounder, marginal structure model, cox model

## Abstract

**Objective:** Few studies estimated the effect of leisure-time physical activity (LTPA) on cardiovascular disease (CVD) risk among hypertensive patients in a longitudinal cohort. This study aims to evaluate the association between LTPA and CVD in a longitudinal management cohort of hypertensive patients.

**Methods:** A total of 58,167 hypertensive patients without baseline CVD from a longitudinal cohort were included in this study. LTPA and other covariates were measured at the follow-up four times annually. The primary outcome was CVD events. The association between LTPA and CVD was assessed by the marginal structure model (MSM) and Cox model with adjustment for age, gender, body mass index (BMI), smoking, drinking, diabetes, hyperlipidemia, and antihypertensive medication. The restricted cubic spline and segmented regression were used to assess the dose–response relationship between LTPA and CVD.

**Results:** We recorded 16,332 CVD events; crude incidence of CVD were 89.68, 80.39, 62.64, and 44.04 per 1,000 person-years for baseline 0, 1–150, 151–300, and >300 min/week LTPA, respectively. Compared with inactive LTPA, the adjusted hazard ratios (HRs) estimated by Cox model and MSM-Cox model for CVD associated with 1–150,151–300, and 300 min/week LTPA were 0.85 (95% CI, 0.83–0.88), 0.67 (95% CI, 0.64–0.71), 0.47 (95% CI, 0.44–0.51), and 0.83 (95% CI, 0.76–0.91), 0.58 (95% CI, 0.52–0.63), and 0.39 (95% CI, 0.35–0.44), respectively. Per 60 min/week increase in LTPA was associated with a 13% reduction in CVD risk. LTPA breakpoint was 417 min/week for CVD. Before and after the break-point, the slopes of the piecewise-linear relationship between LTPA and CVD risk were −0.0017 and −0.0003, respectively.

**Conclusion:** LTPA was more strongly associated with the CVD risk than that estimated by conventional analyses based on baseline LTPA; 417 min/week is a breakpoint, after which the incremental health benefits on CVD prevention obtained from the increase in LTPA are much less than before.

## Introduction

The 2019 American College of Cardiology (ACC)/American Heart Association (AHA) Guideline on the Primary Prevention of Cardiovascular Disease (1) and the 2018 Physical Activity Guidelines for Americans (2) both recommended at least 150 min/week of moderate–intensity physical activity (PA) or 75 min/week of vigorous-intensity PA to stay healthy. Many previous studies have suggested that the PA could reduce the risk of cardiovascular disease (CVD) (3–5) and weaken its risk factors, such as diabetes, hypertension, and obesity (6–8). However, few studies have assessed the association between leisure-time PA (LTPA) and CVD among hypertensive patients in a longitudinal cohort with time-dependent confounders.

Previous studies used a single measurement value to evaluate LTPA over the study period and assumed it was constant, such as baseline LTPA or the average of several LTPA measurements over a certain period. However, LTPA is not a static lifestyle but a long-term dynamic process, which may change over time and be affected by many factors. For example, the blood pressure (BP) of hypertensive patients varies over time during the study period, and BP is not only associated with CVD risk but also affects LTPA. Meanwhile, LTPA also has an impact on BP at the next time point (9–11). In the above case, we regard BP as a time-dependent confounder. A diagram of the time-dependent confounder is shown in Supplementary Figure 1. In the presence of time-dependent confounders, conventional analysis (such as hierarchical analysis, regression analysis) without appropriate accounting for dynamic interaction between time-varying LTPA and time-dependent confounders is prone to biased estimates (12). Therefore, we should adjust for time-dependent confounders when assessing the association between LTPA and CVD risk. Robin et al. proposed the marginal structure model (MSM), which was developed for causal inference of time-varying treatments in observational longitudinal studies with the presence of time-dependent confounders (12). The MSM uses the inverse probability of treatment weights (IPTWs) to create a virtual population and eliminates the influence of time-dependent confounders on the association estimation. To our knowledge, no previous study has used this model to estimate the association between physical activity and CVD in a longitudinal cohort.

The primary objective of this study was to estimate the effect of LTPA on CVD risk in a longitudinal cohort of hypertensive patients and to demonstrate the merits of MSM by comparing it with Cox proportional hazards model. The secondary goal of this study is to investigate the piecewise-linear relationship between LTPA and CVD risk and to explore whether there are break-points of the LTPA where the increase rate in health benefits changes significantly.

## Methods

### Study Cohort

The data for this study were collected from a community-based hypertension longitudinal management cohort in Shandong Province. Patients diagnosed with hypertension between June 2010 and October 2017 were included in the cohort. The patients in this cohort were followed up face to face four times every year. LTPA and other covariates were recorded at each follow-up. We included patients who met the following criteria: (1) no previous history of CVD events; (2) participants without abnormal BP [systolic blood pressure (SBP) ≥200 mmHg or SBP <80 mgHg or diastolic blood pressure (DBP) ≥140 mmHg or DBP <40 mmHg] at any follow-up; (3) no covariates missing; (4) participants with age ranging from 30 to 80 years; and (5) at least three follow-ups. Supplementary Figure 2 presented the flowchart of enrollment. The study protocol was approved by the ethics committee of the School of Public Health, Shandong University.

### Study Outcome

The primary outcome was CVD events, which included coronary heart disease (CHD) and cerebrovascular diseases (CBD). The International Classification of Diseases, 10th Revision (ICD-10) clinical codes were used to identify outcomes. The ICD-10 codes for CHD included I20, I21, I22, I23, I24, and I25. The ICD-10 codes for CBD included I60, I61, I62, I63, I64, I65, I66, and I67. To determine whether participants suffered from CVD events during the study period, the participants' unique identification card numbers were used to matched CVD events in Shandong medical insurance database, hospital electronic medical record data, and Shandong death registration data for every participant. The earliest record date of CHD and CBD in the above database was defined as the diagnosis date of CHD and CBD, respectively. CVD diagnosis date was defined as the earlier date of CHD and CBD diagnosis. Participants who did not matched with CVD events in the three databases were regarded as non-CVD.

### LTPA

In this study, LTPA refers to activities that are taken consciously to strengthen physical fitness. Light to moderate intensity PA includes brisk walking, jogging/running, square dancing, bicycling, and table tennis. In every follow-up visit, the doctors of the community health service center obtained the LTPA frequency (times/week) and duration (minutes/time) of patients in the last 3 months through inquiries. We multiply the frequency (times/week) and duration (minutes/time) to obtain the weekly LTPA time (minutes/week), which was used to measure LTPA levels of hypertensive patients. According to the LTPA levels (150, 300 min/week) recommended by PA guidelines (9–11), the total participants have been categorized into four groups: 0, 1–150, 151–300, and >300 min/week. This study used two forms of LTPA measurement [per increase of 60 min/week; 4 LTPA categories (0, 1–150, 151–300, >300 min/week)] to estimate the association between LTPA and CVD risk.

### Covariates

The covariates include age, sex, SBP, DBP, body mass index (BMI), smoking, drinking, diabetes, hyperlipidemia, and antihypertensive medication (yes/no). Height and weight were measured when patients were wearing light clothing without shoes. BMI is calculated as weight (kg) divided by the square of height (m). After 5 min of rest, the right upper arm blood pressure, including SBP and DBP, was measured in a sitting position by doctors. Smoking and drinking were defined as smoking at least one cigarette a day and drinking alcohol at least once a week on average, respectively. The diagnosis records of patients in Shandong Provincial Medical insurance database and electronic medical records of hospitals were used to determine whether the patients had diabetes and hyperlipidemia. Antihypertensive medication use is defined as the cumulative use of any antihypertensive drug for more than 3 months. Antihypertensive medication included alpha-blocker, angiotensin II receptor blocker, angiotensin-converting enzyme inhibitor, beta-blocker, calcium-channel blocker, diuretics, or any combination of the above classes of medications. A list of antihypertensive medications and Anatomical Therapeutic Chemical is shown in Supplementary Table 1.

### Statistical Analysis

If the continuous variables follow normality, they were presented as mean and standard deviations (SDs), and the analysis of variance (ANOVA) was used for comparison between groups; otherwise, they were presented as medians with interquartile range (IQR), and the Kruskal–Wallis *H* test was applied for intergroup comparison. Categorical variables were summarized as frequencies and proportions; the comparison between groups was carried out using the chi-square test.

A multivariable Cox proportional hazards regression model was used to examine the association between baseline LTPA and CVD risk with adjustment for baseline covariates including age, gender, BMI, smoking, drinking, diabetes, hyperlipidemia, and antihypertensive medication. The marginal structural Cox model (MSM-Cox) was used to examine the association of LTPA with CVD risk with adjustment for time-dependent confounders. First, the model estimates the inverse probability of receiving a specific intervention given the baseline and time-dependent confounders. Then, the IPTW is used to create a virtual population in which the confounding effects are eliminated by weighting and the effect of treatment remains unchanged. In this pseudo-population, Cox proportional hazards model was applied to estimate the association of treatment and outcomes. More details of the MSM-Cox refers to this artile. (13). LTPA was included in the model in the form of continuous variables (per increase of 60 min/week) and categorical variables (0, 1–150, 151–300, >300 min/week). The corresponding hazard ratios (HRs) estimated by the MSM should be interpreted as the risk of CVD for someone whose LTPA increase by 60 min/week and the risk of CVD for someone whose LTPA remained in a particular LTPA category over the study period, respectively. We also conducted sensitivity analyses to investigate the association in different subpopulations stratified by age (≤60, 60–70, ≥70 years), gender (men, women), BMI (<24, 24–27, and >27 kg/m^2^), diabetes mellitus (yes/no), hyperlipidemia (yes/no), smoking (yes/no), drinking (yes/no), antihypertensive medication (yes/no), and test for interactions of LTPA with covariates by adding interactions terms to the model.

In addition, this study conducted two analyses to explore the dose–response relationship and piecewise-linear relationship between LTPA and CVD risks. The first analysis used MSM with restricted cubic splines to fit the nonlinear association between LTPA and CVD risk and used ANOVA to test for nonlinearity. In the second analysis, we used the segmented regression to fit the piecewise-linear relationship between LTPA and CVD risk and to explore whether there are breakpoints of the LTPA time at which the increased rate of health benefits changes significantly.

All data analyses were performed by R (version 3.5.5;) with “ipw” packages and “segmented” packages; *P* < 0.05 was considered significant.

## Results

A total of 58,167 patients (46.4% were men) were included in this study, the median baseline age was 62.2 (IQR, 53.7, 69.0) years. The median baseline SBP and DBP were 145.0 (IQR, 138.0, 154.0) mmHg and 88.0 (IQR, 80.0, 91.0) mmHg, respectively. The participants in the four baseline LTPA categories (0, 1–150, 151–300, >300 min/week) accounted for 41.6, 36.3, 15.8, and 6.4%, respectively (Table 1). While the corresponding values were 33.4, 38.8, 19.4, and 8.4% at all person-quarters (Supplementary Table 2). Participants had 15.2 (range, 3–37) times of follow-up records on average. The median follow-up time was 4.0 years (25th to 75th percentile, 2.0–4.8 years). A total of 16,332 CVD, 11,005 CHD, and 11,021 CBD were identified during the follow-up period, with an incidence density of 78.8 (95% CI, 77.7–80.0), 50.2 (95% CI, 49.3–51.2), and 50.3 (95% CI, 49.4–51.2) per 1,000 person-years, respectively. Among the participants in the four baseline LTPA categories (0, 1–150, 151–300, >300 min/week), the crude incidence of CVD events was 89.7 (95% CI, 87.7–91.6), 80.4 (95% CI, 78.5–82.3), 62.6 (95% CI, 60.0–65.3), and 44.0 (95% CI, 40.6–47.5) per 1,000 person-years, respectively. Estimated 5-year cumulative incidence of CVD for participants in four LTPA categories (0, 1–150, 151–300, >300 min/week) were 35.4, 32.2, 25.2, and 19.0%, respectively. The cumulative incidence functions for CVD, CHD, and CBD by baseline LTPA categories are shown in Supplementary Figure 3.

**Table 1:** Characteristics of the study population by baseline physical activity level.

**Characteristic**	**Overall (*n* = 58,167)**	**Physical activity time (min/week)**				
		**0 (*n* = 24,168)**	**1–150 (*n* = 21,114)**	**150–300 (*n* = 9,166)**	**>300 (*n* = 3,719)**	***P***
**Age, years [M (IQR)]**	62.2 [53.7, 69.0]	61.6 [52.4, 68.7]	62.4 [54.1, 69.3]	52.3 [54.4, 68.9]	64.7 [57.5, 69.3]	<0.001
**Gender**, ***n*****(%)**						
Women	31,200 (53.6)	12,989 (53.7)	11,407 (54.0)	4,909 (53.6)	1,895 (51.0)	
Men	26,967 (46.4)	11,179 (46.3)	9,707 (46.0)	4,257 (46.4)	1,824 (49.0)	0.007
**BMI, kg/m**^**2**^ **[M (IQR)]**	25.0 [23.4, 27.1]	24.9 [23.3, 27.1]	24.8 [23.3, 27.0]	25.3 [23.5, 27.3]	25.8 [23.9, 27.8]	<0.001
**SBP, mmHg [M (IQR)]**	145.0 [138.0, 154.0]	145.0 [138.0, 153.0]	146.0 [140.0, 155.0]	144.0 [138.0, 151.0]	140.0 [134.0, 150.0]	<0.001
**DBP, mmHg [M (IQR)]**	88.0 [80.0, 91.0]	88.0 [80.0, 90.0]	89.0 [80.0, 92.0]	88.0 [80.0, 91.0]	88.0 [80.0, 90.0]	<0.001
**Diabetes mellitus**, ***n*** **(%)**						
No	49,436 (85.0)	20,798 (86.1)	18,027 (85.4)	7,537 (82.2)	3,074 (82.7)	
Yes	8,731 (15.0)	3,370 (13.9)	3,087 (14.6)	1,629 (17.8)	645 (17.3)	<0.001
**Smoker**, ***n*** **(%)**						
No	47,178 (81.1)	19,538 (80.8)	17,083 (80.9)	7,422 (81.0)	3,135 (84.3)	
Yes	10,989 (18.9)	4,630 (19.2)	4,031 (19.1)	1,744 (19.0)	584 (15.7)	<0.001
**Drinker**, ***n*** **(%)**						
No	49,356 (84.9)	20,792 (86.0)	17,741 (84)	7,704 (84.0)	3,119 (83.9)	
Yes	8,811 (15.1)	3,376 (14.0)	3,373 (16.0)	1,462 (16.0)	600 (16.1)	<0.001
**Hyperlipidemia**, ***n*****(%)**						
No	55,801 (95.9)	23,203 (96.0)	20,246 (95.9)	8,767 (95.6)	3,585 (96.4)	
Yes	2,366 (4.1)	965 (4.0)	868 (4.1)	399 (4.4)	134 (3.6)	0.22
**Medication**, ***n*** **(%)**						
No	26,149 (45.0)	10,934 (45.2)	9,766 (46.3)	4,071 (44.4)	1,378 (37.1)	
Yes	32,018 (55.0)	13,234 (54.8)	11,348 (53.7)	5,095 (55.6)	2,341 (62.9)	<0.001

*Data are presented as mean ± SD, median (range) or percentage. BMI, body mass index; SBP, systolic blood pressure; DBP, diastolic blood pressure; LTPA, leisure time physical activity*.

**Figure 1:**
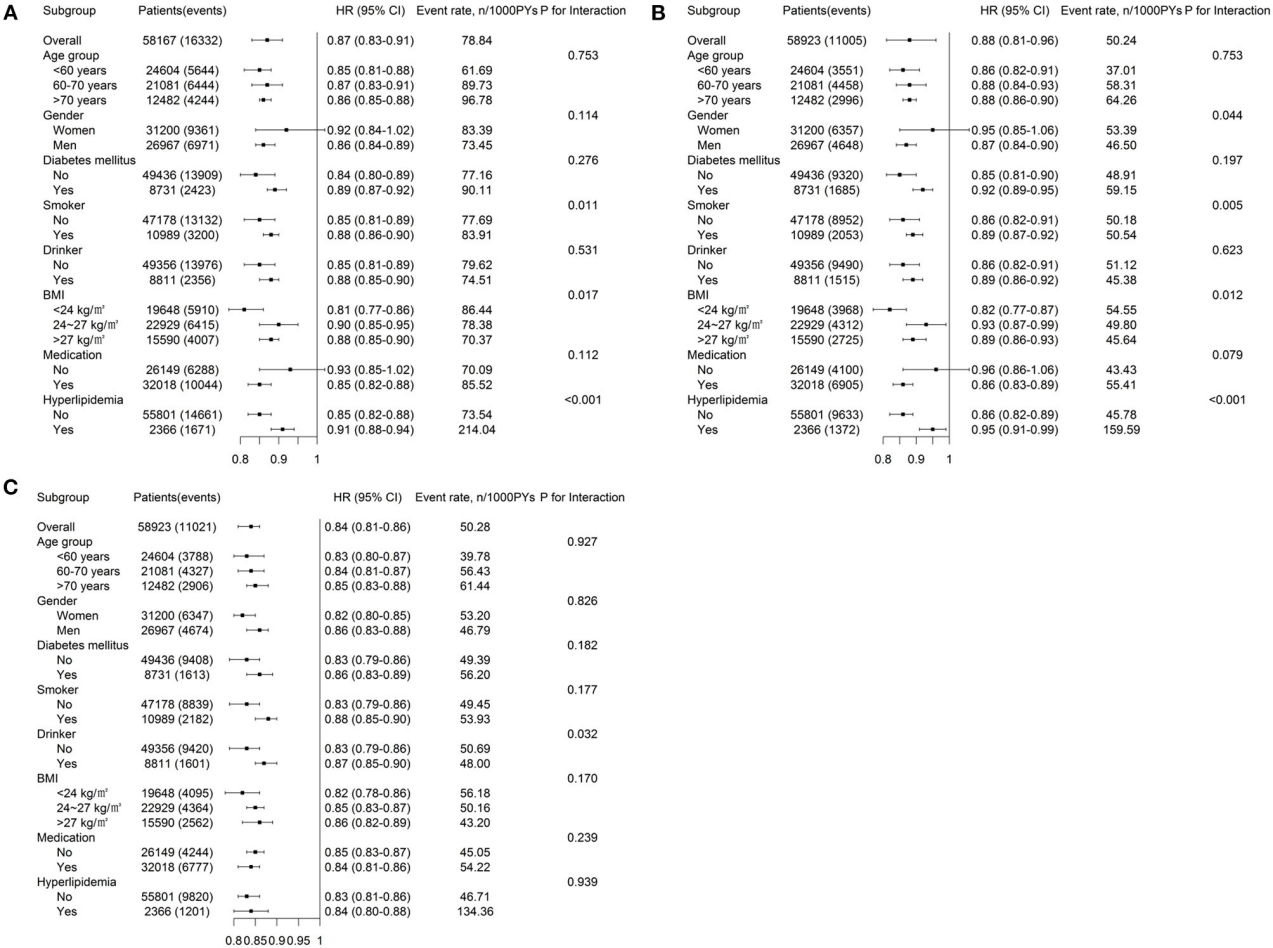
Adjusted HRs (95% CIs) for **(A)** CVD, **(B)** CHD, and **(C)** CBD associated with time-updated LTPA estimated by the MSM. HRs, hazard ratios; CVD, cardiovascular disease; CHD, coronary heart diseases; CBD, cerebrovascular diseases; BMI, body mass index; LTPA, leisure time physical activity; PYs, person-years; *P*-value is for interactions of time-updated LTPA with these covariates.

After multivariable adjustment in the Cox model, compared with participants in baseline LTPA 0 min/week category, the adjusted HRs associated with 1–150, 151–300, and >300 min/week LTPA categories were 0.85 (95% CI, 0.83–0.88), 0.67 (95% CI, 0.64–0.71), and 0.47 (95% CI, 0.44–0.51) for CVD, respectively. However, the analysis of the MSM-Cox with adjustment for time-dependent confounders showed that the corresponding adjusted HRs were 0.83 (95% CI, 0.77–0.91), 0.58 (95% CI, 0.53–0.63), and 0.39 (95% CI, 0.35–0.44), respectively. Table 2 summarizes the adjusted HR estimated by the MSM-Cox and Cox models. We also used LTPA as a continuous variable to conduct the Cox model and MSM-Cox analysis (Table 3). The Cox model showed that per 60 min/week LTPA increase was significantly associated with a 9.00% reduction of CVD risk, while the corresponding value estimated by MSM-Cox was 13%. The stratified analyses showed that there was no significant difference in the strength of association between LTPA and CVD among subgroups stratified by age, gender, diabetes, drinking, and antihypertensive medication (Figure 1, Supplementary Tables 3–8). In the subgroups of nonsmokers, no hyperlipidemia, or BMI <24 kg/m^2^, the protective effect of LTPA on CVD was higher than that of other corresponding subgroups.

**Table 2:** Adjusted hazards ratios for risk of CVD, CHD, CBD, and PAF associated with PAT.

**Characteristics**	**Physical activity Time**				***P* for trend**^**‡**^	**Adjusted PAF (95%CI)**
	**0**	**1–150**	**150–300**	**>300**		
Num of Participants	24,168	21,114	9166	3719		
**CVD**						
Events	7,369	6,294	2,063	606		
Total person-years (PYs)	82,168	78,296	32,936	13,759		
Crude incidence, *n*/1,000 PYs	89.68	80.39	62.64	44.04		
Adjusted HR estimated by Cox model^*^	1.00	0.85 (0.83–0.88)	0.67 (0.64–0.71)	0.47 (0.44–0.51)	<0.001	11.32 (10.00–12.62)
Adjusted HR estimated by MSM-Cox^†^	1.00	0.83 (0.77–0.91)	0.58 (0.53–0.63)	0.39 (0.35–0.44)	<0.001	15.30 (11.71–18.94)
**CHD**						
Events	4,822	4,372	1,364	447		
Total person-years (PYs)	87,749	82,735	34,459	14,086		
Crude incidence, *n*/1,000 PYs	54.95	52.84	39.58	31.73		
Adjusted HR estimated by Cox model^*^	1.00	0.90 (0.86–0.94)	0.69 (0.65–0.74)	0.55 (0.50–0.61)	<0.001	8.88 (7.28–10.49)
Adjusted HR estimated by MSM-Cox^†^	1.00	0.87 (0.77–0.98)	0.58 (0.52–0.66)	0.45 (0.39–0.53)	<0.001	13.50 (8.41–18.55)
**CBD**						
Events	5,149	4,179	1,344	349		
Total person-years (PYs)	87,177	83,464	34,400	14,153		
Crude incidence, *n*/1,000 PYs	59.06	50.07	39.07	24.66		
Adjusted HR estimated by Cox model^*^	1.00	0.81 (0.77–0.84)	0.64 (0.60–0.68)	0.41 (0.37–0.46)	<0.001	13.97 (12.33–15.55)
Adjusted HR estimated by MSM-Cox^†^	1.00	0.78 (0.71–0.86)	0.54 (0.49–0.60)	0.32 (0.28–0.37)	<0.001	18.35 (14.27–22.40)

*LTPA, leisure time physical activity; PYs, person-years; CVD, cardiovascular diseases; CHD, coronary heart disease; CBD, cerebrovascular diseases; HR, Hazard ratio; PAF, adjusted population attributable fraction*.

* *Using the Cox model with adjustment for baseline covariates included age, gender, BMI, smoking, drinking, exercise, diabetes, hyperlipidemia, and medication*.

† *Using the MSMs with adjustment for time-updated covariates as above and time-depended confounding*.

‡ *P-value for trends across the categories of LTPA; all tests were two-tailed*.

**Table 3:** Multivariable associations of outcomes with LTPA increase of 60 min/week estimated by MSM-Cox and Cox model.

**Age group**	Cox model^*^		**MSM-Cox**^**†**^	
	**OR**	**95% CI**	**OR**	**95% CI**
**CVD**				
≤60	0.92	0.91–0.94	0.85	0.81–0.88
60–70	0.91	0.89–0.92	0.87	0.83–0.91
>70	0.91	0.89–0.92	0.86	0.85–0.88
Overall	0.91	0.91–0.92	0.87	0.83–0.91
**CHD**				
≤60	0.93	0.92–0.95	0.86	0.82–0.91
60–70	0.92	0.90–0.93	0.88	0.84–0.93
>70	0.92	0.91–0.94	0.88	0.86–0.90
Overall	0.93	0.92–0.93	0.88	0.83–0.93
**CBD**				
≤60	0.91	0.89–0.92	0.83	0.80–0.87
60–70	0.89	0.88–0.90	0.84	0.81–0.87
>70	0.89	0.87–0.91	0.85	0.83–0.88
Overall	0.90	0.89–0.91	0.84	0.81–0.86

*CVD, cardiovascular diseases; CHD, coronary heart disease; CBD, cerebrovascular diseases; HR, Hazard ratio; LTPA, leisure time physical activity*.

* *Using the Cox model with adjustment for baseline covariates included age, gender, BMI, smoking, drinking, exercise, diabetes, hyperlipidemia, and medication*.

† *Using the MSMs with adjustment for time-varying covariates as above and time-depended confounding*.

**Figure 2:**
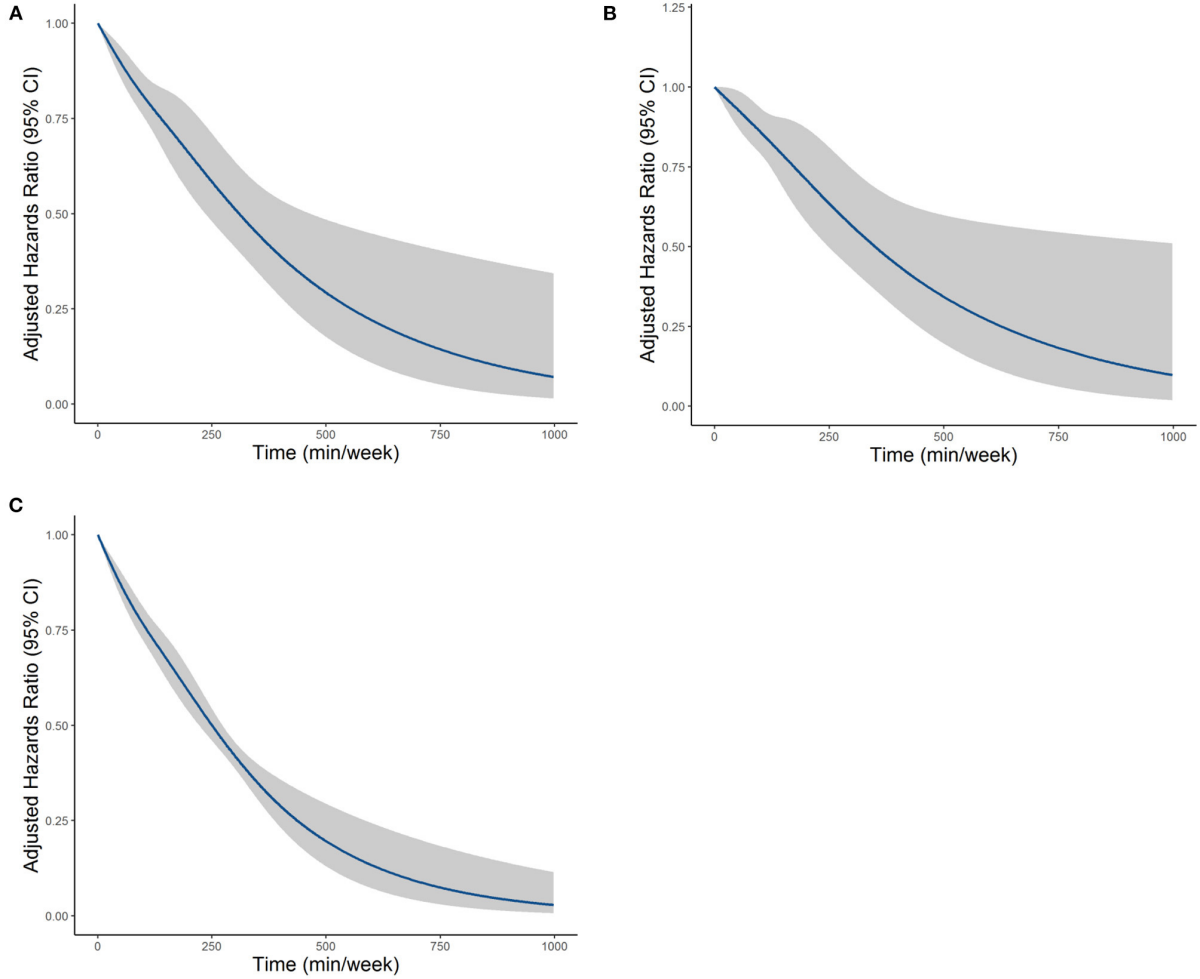
The dose–response relationships between LTPA with **(A)** CVD, **(B)** CHD, and **(C)** CBD, which were estimated by the MSM-Cox with restricted cubic splines. CVD, cardiovascular disease; CHD, coronary heart diseases; CBD, cerebrovascular diseases; LTPA, leisure time physical activity.

**Figure 3:**
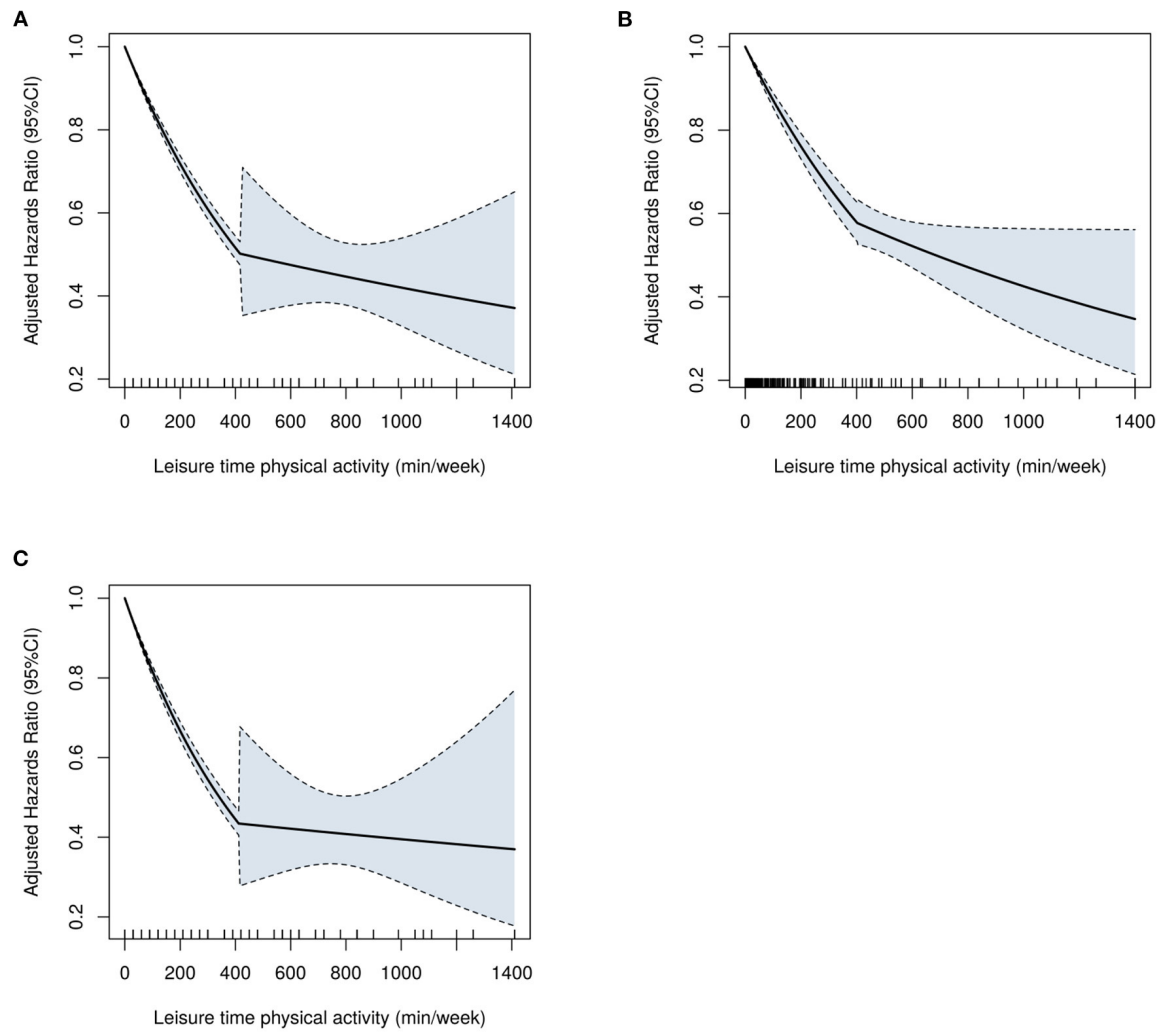
The piecewise-linear relationships between LTPA with **(A)** CVD, **(B)** CHD, and **(C)** CBD, which were estimated by the segmented regression. CVD, cardiovascular disease; CHD, coronary heart diseases; CBD, cerebrovascular diseases; LTPA, leisure time physical activity.

The MSM with the five knots restricted cubic spline indicated that there was a nonlinearity relationship between LTPA (as a continuous variable) with CVD (*P* < 0.001 for nonlinearity test), CHD (*P* < 0.001 for nonlinearity test), and CBD (*P* < 0.001 for nonlinearity test) (Figure 2). Figure 3 shows the piecewise-linear relationships between LTPA and CVD risk. The estimation of LTPA breakpoint was 417 min/week for CVD. The parameters of the piecewise-linear relationship between CVD events and LTPA are summarized in Supplementary Table 9. Before the breakpoint, the protective effect of LTPA on CVD increased rapidly (slope = −0.0017), but after the breakpoint, the benefits from LTPA for protection of CVD increased slowly (slope = −0.0003).

## Discussion

In this community-based longitudinal cohort of hypertensive patients, we evaluated the association between LTPA and CVD events using the Cox model and MSM-Cox model. Our study showed that LTPA had a stronger association with CVD events after appropriately adjusting for time-dependent confounders than that estimated by the Cox model with baseline LTPA. Our study also explored the piecewise-linear relationship between LTPA and CVD events and estimated breakpoints of LTPA. To the best of our knowledge, this is the first study to use MSM to evaluate the protective effect of LTPA on CVD in a longitudinal cohort with time-dependent confounders.

Many previous studies have examined the association between LTPA and CVD events and supported an inverse relationship between them (6, 14–18). However, few studies have explored the association and dose–response relationship between LTPA and CVD risk among hypertensive patients in a longitudinal cohort. Lee and Shiroma (15) reviewed the epidemiological studies on PA and cardiovascular health and found that compared with the least active subjects, the most active subjects had a 30–35% reduction of CHD risk and about 40% reduction of CVD risk. Scarborough et al. (14) found that participants achieving recommended PA levels (150 min/week) had a 23% lower risk for CVD mortality and a 17% lower risk for CVD incidence than inactive participants. Our study found that the protective effect of LTPA on the risk of CVD/CHD in hypertensive patients is greater than the results reported in the above studies. There are two main aspects of physical activity on health protective effects. The first is to improve fitness and physical function (19). The second aspect is that PA regulates the harmful relationship between cardiovascular disease and its risk factors (20). Many studies were consistent with the reverse relationship between LTPA and risk factors of CVD, such as hypertension (21), diabetes (22), obesity (8), and C-reactive protein (23).

In this study, the strength of the association between LTPA and CVD risk estimated by the Cox model was lower than that estimated by the MSM-Cox model. This finding may reflect shortcomings in previous research methods. What we were interested in was the effect of LTPA on the risk of CVD when LTPA is constant at a certain level throughout the study period, but the Cox model could not reply to this question well. Most previous studies have relied on LTPA measurements in a single time point or the average of some LTPA measurements over a certain period for association study and assumed that it was constant (6, 14–18). However, LTPA is not a static but a long-term and dynamic process that may vary over time, and BP or other factors may reduce or increase the LTPA at the next time point. The Cox models could not adequately handle time-dependent confounders. The MSM has been used in several previous studies (24–26) to adequately handle time-dependent confounders. Based on the comparison of analysis results between the Cox models and the MSM in this study, it is reasonable to speculate that the effect of LTPA on the prevention of CVD was probably underestimated in previous studies.

The association between very high PA levels and health benefits remains highly controversial (27, 28). Some studies suggested a reverse J-shape (29) or even a U-shaped association in which CVD events increase among the participants with very high PA levels (30, 31). Other studies (18, 32) suggested that very high LTPA had beneficial effects on health. In this study, we conducted two analyses to explore the dose–response relationship between LTPA and CVD risks. Both analyses showed that very high LTPA had a protective effect on CVD. In addition, we also found that there may be breakpoints of the LTPA time at which the increased rate of health benefits changes significantly. Before the breakpoint, the health benefits of LTPA on CVD prevention increased rapidly with the increase in time spent in LTPA, but after the breakpoint, the increased rate of the health benefits decreased significantly. Similar dose–response relationships were also found in the Zhao et al. (32) study.

Our study has several strengths. First, the data were collected from a relatively large-size community-based hypertension management longitudinal cohort that could represent the general community population with hypertension in Shandong Province. Second, we used the MSM-Cox model to assess the association between LTPA and CVD with adjustment for time-dependent confounders and explored the piecewise-linear relationship between LTPA and CVD. Our study also has some limitations. A major limitation of this study is that LTPA was measured by patients' self-reported information, which may be affected by recall bias. Compared with objective measurement of PA, the criterion validity of self-reported LTPA is relatively limited (33). Second, participants included in this study had to have complete data and at least three times study visits, which may lead to selection bias. In addition, the lack of data on PA at work and during transportation, as well as data on sedentary behaviors, is also a limitation of the study.

## Conclusions

Our study emphasized that the effect of LTPA on the prevention of CVD in hypertensive patients was stronger than that estimated in conventional analyses based on baseline LTPA. Four hundred seventeen min per week is a breakpoint of LTPA; after achieving the breakpoint, the incremental health benefits of CVD prevention obtained from the increase in LTPA were much less than before. This should be taken into account when developing PA guidelines for hypertensive patients.

## Data Availability Statement

The data used to support the findings of this study are available from the corresponding author upon request.

## Ethics Statement

The studies involving human participants were reviewed and approved by the ethics committee of the School of Public Health, Shandong University. The patients/participants provided their written informed consent to participate in this study.

## Author Contributions

JL, ZZ, and FX designed the study, performed data analyses, and had full access to all the data in the study. JL drafted the manuscript. FX and SS revising the manuscript critically for important intellectual content. All authors contributed to the article and approved the submitted version.

## Conflict of Interest

The authors declare that the research was conducted in the absence of any commercial or financial relationships that could be construed as a potential conflict of interest.
