# Supplementary material for: Leisure-Time Physical Activity and Cardiovascular Disease Risk Among Hypertensive Patients: A Longitudinal Cohort Study
Source: Front Cardiovasc Med. 2021 May 28;8:644573. doi: 10.3389/fcvm.2021.644573 (PMC8193126; doi:10.3389/fcvm.2021.644573)
Supplement: Supplementary file 1 [file Data_Sheet_1.docx]

| Table S1 A list of Antihypertensive medication and Anatomical Therapeutic Chemical Code | | | |
| --- | --- | --- | --- |
| ATC | Drug Name | ATC | Drug Name |
| C08CA05 | nifedipine | C08CA02 | felodipine |
| C09AA02 | enalapril | C08DB01 | diltiazem |
| C07AB02 | metoprolol | C07AB05 | betaxolol |
| C03AH02 | hydroflumethiazide, combinations | C07AG02 | carvedilol |
| C09AA01 | captopril | C09AA16 | imidapril |
| C09CA04 | irbesartan | C09CA06 | candesartan |
| C03BA11 | indapamide | C09AA04 | perindopril |
| C02AA52 | reserpine, combinations | C08CA09 | lacidipine |
| C08CA08 | nitrendipine | C07AA05 | propranolol |
| C08CA06 | nimodipine | C09CA01 | losartan |
| C09CA07 | telmisartan | C08CA15 | benidipine |
| C07AB07 | bisoprolol | C09AA07 | benazepril |
| C03AA03 | hydrochlorothiazide | C09AA03 | lisinopril |
| C08CA01 | amlodipine | C07AB03 | atenolol |
| C09CA03 | valsartan | C03DB02 | triamterene |
| C09DB01 | valsartan and amlodipine | |  |

| Table S2. Characteristics of the Study Population by Baseline LTPA Levels | | | | | | |
| --- | --- | --- | --- | --- | --- | --- |
| Characteristic | Overall (n=886940) | Physical Acitivity Time (min/week) | | | | |
|  |  | 0 (n=295920) | 1-150 (n=344456) | 150-300 (n=172097) | >300 (n=74467) | *P* |
| Age, years (M [IQR]) | 61.8 [53.5, 68.9] | 61.8 [52.8, 69.2] | 61.7 [53.5, 69.1] | 61.6 [53.9, 68.5] | 63.1 [55.9, 68.7] | <0.001 |
| Gender, n (%) |  |  |  |  |  |  |
| Women | 480282 (54.2) | 160066 (54.1) | 189163 (54.9) | 92798 (53.9) | 38217 (51.3) |  |
| Men | 406658 (45.8) | 135854 (45.9) | 155293 (45.1) | 79261 (46.1) | 36250 (48.7) | <0.001 |
| BMI, kg/m2 (M [IQR]) | 25.0 [23.3, 27.1] | 24.8 [23.2, 27.0] | 24.8 [23.2, 26.8] | 25.2 [23.5, 27.3] | 25.8 [23.9, 27.9] | <0.001 |
| SBP, mmHg (M [IQR]) | 138.0 [132.0, 145.0] | 138.0 [132.0, 146.0] | 138.0 [132.0, 146.0] | 136.0 [130.0, 142.0] | 136.0 [130.0, 140.0] | <0.001 |
| DBP, mmHg (M [IQR]) | 84.0 [80.0, 88.0] | 84.0 [80.0, 89.0] | 84.0 [80.0, 88.0] | 82.0 [80.0, 88.0] | 82.00 [80.0, 80.0] | <0.001 |
| Diabetes, n (%) |  |  |  |  |  | <0.001 |
| No | 770637 (86.9) | 259485 (87.7) | 300042 (87.1) | 147258 (85.6) | 63814 (85.7) |  |
| Yes | 116303 (13.1) | 36435 (12.3) | 44414 (12.9) | 24801 (14.4) | 10653 (14.3) |  |
| Smoker, n (%) |  |  |  |  |  | <0.001 |
| No | 724147 (81.6) | 236680 (80) | 281179 (81.6) | 141933 (82.5) | 64317 (86.4) |  |
| Yes | 162793 (18.4) | 59240 (20.0) | 63277 (18.4) | 30126 (17.5) | 10150 (13.6) |  |
| Drinker, n (%) |  |  |  |  |  | <0.001 |
| No | 758301 (85.5) | 252660 (85.4) | 293448 (85.2) | 146853 (85.4) | 65302 (87.7) |  |
| Yes | 128639 (14.5) | 43260 (14.6) | 51008 (14.8) | 25206 (14.6) | 9165 (12.3) |  |
| Hyperlipidemia, n (%) |  |  |  |  |  | <0.001 |
| No | 853348 (96.2) | 285051 (96.3) | 331111 (96.1) | 165656 (96.3) | 71492 (96.0) |  |
| Yes | 33592 (3.8) | 10869 (3.7) | 13345 (3.9) | 6403 (3.7) | 2975 (4.0) |  |
| antihypertensive Medication, n (%) <0.001 | | | | | | |
| No | 351664 (39.6) | 117117 (39.6) | 145370 (42.2) | 65436 (38) | 23703 (31.8) |  |
| Yes | 535276 (60.4) | 178803 (60.4) | 199086 (57.8) | 106623 (62.0) | 50764 (68.2) |  |

Data are presented as mean (SD), median (range) or percentage. BMI, body mass index; SBP, systolic blood pressure; DBP, diastolic blood pressure; LTPA, leisure time physical activity;

| Table S3 Multivariable associations between CVD and time-updated LTPA estimated by the MSM within subgroups | | | | | | | |
| --- | --- | --- | --- | --- | --- | --- | --- |
| Characteristics | PAT | N | Person-years | Events | Event rates (1000PYs) | HR (95%CI) | P for Interaction |
|  |  |  |  |  |  |  |  |
| Age group | |  |  |  |  |  | 0.551 |
| <60 | 0 | 10789 | 38371 | 2683 | 69.92 |  |  |
|  | 0-150 | 8837 | 34116 | 2040 | 59.80 | 0.94 (0.79-1.12) |  |
|  | 150-300 | 3797 | 14276 | 710 | 49.73 | 0.72 (0.60-0.86) |  |
|  | >300 | 1181 | 4732 | 211 | 44.59 | 0.51 (0.39-0.67) |  |
| 60-70 | 0 | 8385 | 27066 | 2832 | 104.63 |  |  |
|  | 0-150 | 7581 | 26780 | 2529 | 94.44 | 0.79 (0.70-0.90) |  |
|  | 150-300 | 3392 | 11776 | 815 | 69.21 | 0.50 (0.43-0.57) |  |
|  | >300 | 1723 | 6192 | 268 | 43.28 | 0.34 (0.28-0.40) |  |
| >70 | 0 | 4994 | 16731 | 1854 | 110.81 |  |  |
|  | 0-150 | 4696 | 17400 | 1725 | 99.14 | 0.85 (0.76-0.95) |  |
|  | 150-300 | 1977 | 6885 | 538 | 78.14 | 0.59 (0.52-0.67) |  |
|  | >300 | 815 | 2835 | 127 | 44.80 | 0.36 (0.30-0.44) |  |
| Gender |  |  |  |  |  |  | 0.604 |
| Women | 0 | 12989 | 44549 | 4253 | 95.47 |  |  |
|  | 0-150 | 11407 | 42990 | 3641 | 84.69 | 0.81 (0.76-0.87) |  |
|  | 150-300 | 4909 | 17627 | 1145 | 64.96 | 0.56 (0.52-0.61) |  |
|  | >300 | 1895 | 7096 | 322 | 45.38 | 0.39 (0.34-0.44) |  |
| Men | 0 | 11179 | 37619 | 3116 | 82.83 |  |  |
|  | 0-150 | 9707 | 35306 | 2653 | 75.14 | 0.84 (0.7-1.01) |  |
|  | 150-300 | 4257 | 15310 | 918 | 59.96 | 0.57 (0.47-0.70) |  |
|  | >300 | 1824 | 6663 | 284 | 42.62 | 0.39 (0.31-0.49) |  |
| Medication | |  |  |  |  |  | 0.047 |
| No | 0 | 10934 | 36806 | 2794 | 75.91 |  |  |
|  | 0-150 | 9766 | 33927 | 2527 | 74.48 | 0.87 (0.79-0.95) |  |
|  | 150-300 | 4071 | 13919 | 792 | 56.90 | 0.61 (0.55-0.68) |  |
|  | >300 | 1378 | 5063 | 175 | 34.56 | 0.43 (0.36-0.51) |  |
| Yes | 0 | 13234 | 45363 | 4575 | 100.85 |  |  |
|  | 0-150 | 11348 | 44369 | 3767 | 84.90 | 0.83 (0.74-0.95) |  |
|  | 150-300 | 5095 | 19017 | 1271 | 66.83 | 0.56 (0.49-0.64) |  |
|  | >300 | 2341 | 8696 | 431 | 49.56 | 0.37 (0.32-0.43) |  |
| Diabetes mellitus | |  |  |  |  |  | 0.719 |
| No | 0 | 20798 | 72027 | 6319 | 87.73 |  |  |
|  | 0-150 | 18027 | 68609 | 5377 | 78.37 | 0.88 (0.81-0.95) |  |
|  | 150-300 | 7537 | 27896 | 1711 | 61.33 | 0.58 (0.53-0.64) |  |
|  | >300 | 3074 | 11741 | 502 | 42.76 | 0.41 (0.36-0.46) |  |
| Yes | 0 | 3370 | 10142 | 1050 | 103.53 |  |  |
|  | 0-150 | 3087 | 9687 | 917 | 94.66 | 0.76 (0.66-0.86) |  |
|  | 150-300 | 1629 | 5041 | 352 | 69.83 | 0.6 (0.51-0.71) |  |
|  | >300 | 645 | 2018 | 104 | 51.54 | 0.40 (0.32-0.50) |  |
| Smoker |  |  |  |  |  |  | 0.003 |
| No | 0 | 19538 | 66687 | 5940 | 89.07 |  |  |
|  | 0-150 | 17083 | 64032 | 5075 | 79.26 | 0.69 (0.55-0.88) |  |
|  | 150-300 | 7422 | 26755 | 1620 | 60.55 | 0.47 (0.37-0.60) |  |
|  | >300 | 3135 | 11547 | 497 | 43.04 | 0.33 (0.26-0.42) |  |
| Yes | 0 | 4630 | 15481 | 1429 | 92.31 |  |  |
|  | 0-150 | 4031 | 14264 | 1219 | 85.46 | 0.99 (0.85-1.14) |  |
|  | 150-300 | 1744 | 6182 | 443 | 71.66 | 0.72 (0.61-0.85) |  |
|  | >300 | 584 | 2212 | 109 | 49.28 | 0.45 (0.35-0.59) |  |
| Drinker |  |  |  |  |  |  | 0.001 |
| No | 0 | 20792 | 70715 | 6426 | 90.87 |  |  |
|  | 0-150 | 17741 | 65965 | 5323 | 80.69 | 0.80 (0.72-0.88) |  |
|  | 150-300 | 7704 | 27488 | 1725 | 62.75 | 0.54 (0.49-0.60) |  |
|  | >300 | 3119 | 11371 | 502 | 44.15 | 0.37 (0.33-0.42) |  |
| Yes | 0 | 3376 | 11453 | 943 | 82.34 |  |  |
|  | 0-150 | 3373 | 12331 | 971 | 78.74 | 1.03 (0.89-1.18) |  |
|  | 150-300 | 1462 | 5449 | 338 | 62.03 | 0.74 (0.62-0.87) |  |
|  | >300 | 600 | 2388 | 104 | 43.55 | 0.48 (0.33-0.70) |  |
| BMI |  |  |  |  |  |  | 0.200 |
| <24 | 0 | 8336 | 27345 | 2697 | 98.63 |  |  |
|  | 0-150 | 7534 | 27561 | 2368 | 85.92 | 0.85 (0.75-0.96) |  |
|  | 150-300 | 2816 | 9975 | 677 | 67.87 | 0.55 (0.48-0.64) |  |
|  | >300 | 962 | 3493 | 168 | 48.10 | 0.39 (0.33-0.48) |  |
| 24~27 | 0 | 9387 | 32037 | 2846 | 88.83 |  |  |
|  | 0-150 | 8340 | 30850 | 2485 | 80.55 | 0.95 (0.83-1.09) |  |
|  | 150-300 | 3751 | 13593 | 852 | 62.68 | 0.73 (0.62-0.85) |  |
|  | >300 | 1451 | 5362 | 232 | 43.27 | 0.44 (0.36-0.53) |  |
| >27 | 0 | 6445 | 22786 | 1826 | 80.14 |  |  |
|  | 0-150 | 5240 | 19885 | 1441 | 72.47 | 0.81 (0.71-0.91) |  |
|  | 150-300 | 2599 | 9369 | 534 | 57.00 | 0.53 (0.46-0.61) |  |
|  | >300 | 1306 | 4904 | 206 | 42.01 | 0.42 (0.34-0.52) |  |
| Hyperlipidemia | |  |  |  |  |  | 0.001 |
| No | 0 | 23203 | 79237 | 6668 | 84.15 |  |  |
|  | 0-150 | 20246 | 75327 | 5668 | 75.25 | 0.82 (0.76-0.90) |  |
|  | 150-300 | 8767 | 31544 | 1797 | 56.97 | 0.56 (0.51-0.62) |  |
|  | >300 | 3585 | 13245 | 528 | 39.86 | 0.36 (0.32-0.41) |  |
| Yes | 0 | 965 | 2932 | 701 | 239.09 |  |  |
|  | 0-150 | 868 | 2969 | 626 | 210.85 | 0.87 (0.74-1.03) |  |
|  | 150-300 | 399 | 1393 | 266 | 190.95 | 0.68 (0.55-0.82) |  |
|  | >300 | 134 | 514 | 78 | 151.75 | 0.65 (0.43-0.97) |  |

BMI, body mass index; SBP, systolic blood pressure; DBP, diastolic blood pressure; CCB, calcium-channel blocker; ACEI, angiotensin-converting enzyme inhibitor; ARB, angiotensin II receptor blocker; HR, hazard ratio, LTPA, leisure time physical activity.

| Table S4 Multivariable associations between CVD and time-updated LTPA estimated by the MSM within subgroups | | | | | | | |
| --- | --- | --- | --- | --- | --- | --- | --- |
| Characteristics | PAT | N | Person-years | Events | Event rates (1000PYs) | HR (95%CI) | P for Interaction |
|  |  |  |  |  |  |  |  |
| Age group | |  |  |  |  |  | 0.405 |
| <60 | 0 | 10789 | 40532 | 1655 | 40.83 |  |  |
|  | 0-150 | 8837 | 35717 | 1310 | 36.68 | 0.98 (0.78-1.23) |  |
|  | 150-300 | 3797 | 14875 | 437 | 29.38 | 0.74 (0.60-0.92) |  |
|  | >300 | 1181 | 4832 | 149 | 30.84 | 0.63 (0.45-0.87) |  |
| 60-70 | 0 | 8385 | 29206 | 1890 | 64.71 |  |  |
|  | 0-150 | 7581 | 28510 | 1819 | 63.80 | 0.86 (0.70-1.05) |  |
|  | 150-300 | 3392 | 12375 | 549 | 44.36 | 0.50 (0.40-0.62) |  |
|  | >300 | 1723 | 6362 | 200 | 31.44 | 0.39 (0.31-0.49) |  |
| >70 | 0 | 4994 | 18012 | 1277 | 70.90 |  |  |
|  | 0-150 | 4696 | 18509 | 1243 | 67.16 | 0.84 (0.73-0.97) |  |
|  | 150-300 | 1977 | 7210 | 378 | 52.43 | 0.61 (0.52-0.72) |  |
|  | >300 | 815 | 2892 | 98 | 33.89 | 0.39 (0.31-0.49) |  |
| Gender |  |  |  |  |  |  | 0.867 |
| Women | 0 | 12989 | 47732 | 2803 | 58.72 |  |  |
|  | 0-150 | 11407 | 45592 | 2542 | 55.76 | 0.86 (0.80-0.93) |  |
|  | 150-300 | 4909 | 18463 | 777 | 42.08 | 0.59 (0.54-0.65) |  |
|  | >300 | 1895 | 7282 | 235 | 32.27 | 0.46 (0.40-0.53) |  |
| Men | 0 | 11179 | 40018 | 2019 | 50.45 |  |  |
|  | 0-150 | 9707 | 37144 | 1830 | 49.27 | 0.83 (0.63-1.1) |  |
|  | 150-300 | 4257 | 15996 | 587 | 36.70 | 0.55 (0.41-0.74) |  |
|  | >300 | 1824 | 6804 | 212 | 31.16 | 0.42 (0.30-0.59) |  |
| Medication | |  |  |  |  |  | 0.033 |
| No | 0 | 10934 | 39029 | 1731 | 44.35 |  |  |
|  | 0-150 | 9766 | 35731 | 1719 | 48.11 | 0.95 (0.85-1.07) |  |
|  | 150-300 | 4071 | 14490 | 526 | 36.30 | 0.66 (0.57-0.75) |  |
|  | >300 | 1378 | 5155 | 124 | 24.05 | 0.52 (0.42-0.64) |  |
| Yes | 0 | 13234 | 48720 | 3091 | 63.44 |  |  |
|  | 0-150 | 11348 | 47005 | 2653 | 56.44 | 0.84 (0.70-1.00) |  |
|  | 150-300 | 5095 | 19969 | 838 | 41.97 | 0.55 (0.45-0.66) |  |
|  | >300 | 2341 | 8931 | 323 | 36.17 | 0.41 (0.33-0.50) |  |
| Diabetes mellitus | |  |  |  |  |  | 0.637 |
| No | 0 | 20798 | 76898 | 4107 | 53.41 |  |  |
|  | 0-150 | 18027 | 72457 | 3723 | 51.38 | 0.93 (0.83-1.03) |  |
|  | 150-300 | 7537 | 29180 | 1117 | 38.28 | 0.59 (0.53-0.66) |  |
|  | >300 | 3074 | 12010 | 373 | 31.06 | 0.47 (0.41-0.55) |  |
| Yes | 0 | 3370 | 10851 | 715 | 65.89 |  |  |
|  | 0-150 | 3087 | 10279 | 649 | 63.14 | 0.79 (0.66-0.95) |  |
|  | 150-300 | 1629 | 5280 | 247 | 46.78 | 0.64 (0.52-0.79) |  |
|  | >300 | 645 | 2076 | 74 | 35.65 | 0.46 (0.35-0.60) |  |
| Smoker |  |  |  |  |  |  | 0.011 |
| No | 0 | 19538 | 71110 | 3926 | 55.21 |  |  |
|  | 0-150 | 17083 | 67572 | 3566 | 52.77 | 0.69 (0.49-0.96) |  |
|  | 150-300 | 7422 | 27921 | 1095 | 39.22 | 0.46 (0.33-0.65) |  |
|  | >300 | 3135 | 11810 | 365 | 30.91 | 0.36 (0.26-0.50) |  |
| Yes | 0 | 4630 | 16640 | 896 | 53.85 |  |  |
|  | 0-150 | 4031 | 15164 | 806 | 53.15 | 1.01 (0.85-1.20) |  |
|  | 150-300 | 1744 | 6539 | 269 | 41.14 | 0.70 (0.58-0.86) |  |
|  | >300 | 584 | 2276 | 82 | 36.03 | 0.56 (0.41-0.76) |  |
| Drinker |  |  |  |  |  |  | 0.002 |
| No | 0 | 20792 | 75523 | 4241 | 56.16 |  |  |
|  | 0-150 | 17741 | 69703 | 3724 | 53.43 | 0.82 (0.72-0.94) |  |
|  | 150-300 | 7704 | 28772 | 1156 | 40.18 | 0.55 (0.48-0.63) |  |
|  | >300 | 3119 | 11644 | 369 | 31.69 | 0.43 (0.36-0.50) |  |
| Yes | 0 | 3376 | 12226 | 581 | 47.52 |  |  |
|  | 0-150 | 3373 | 13032 | 648 | 49.72 | 1.09 (0.92-1.29) |  |
|  | 150-300 | 1462 | 5687 | 208 | 36.57 | 0.78 (0.63-0.96) |  |
|  | >300 | 600 | 2442 | 78 | 31.94 | 0.56 (0.34-0.93) |  |
| BMI |  |  |  |  |  |  | 0.266 |
| <24 | 0 | 8336 | 29399 | 1776 | 60.41 |  |  |
|  | 0-150 | 7534 | 29285 | 1619 | 55.28 | 0.87 (0.75-1.02) |  |
|  | 150-300 | 2816 | 10470 | 453 | 43.27 | 0.55 (0.46-0.66) |  |
|  | >300 | 962 | 3585 | 120 | 33.47 | 0.46 (0.36-0.57) |  |
| 24~27 | 0 | 9387 | 34264 | 1837 | 53.61 |  |  |
|  | 0-150 | 8340 | 32557 | 1750 | 53.75 | 1.01 (0.87-1.17) |  |
|  | 150-300 | 3751 | 14252 | 556 | 39.01 | 0.75 (0.62-0.92) |  |
|  | >300 | 1451 | 5516 | 169 | 30.64 | 0.48 (0.38-0.59) |  |
| >27 | 0 | 6445 | 24087 | 1209 | 50.19 |  |  |
|  | 0-150 | 5240 | 20893 | 1003 | 48.01 | 0.84 (0.70-1.01) |  |
|  | 150-300 | 2599 | 9738 | 355 | 36.46 | 0.57 (0.47-0.69) |  |
|  | >300 | 1306 | 4986 | 158 | 31.69 | 0.52 (0.40-0.68) |  |
| Hyperlipidemia | |  |  |  |  |  | 0.001 |
| No | 0 | 23203 | 84469 | 4267 | 50.52 |  |  |
|  | 0-150 | 20246 | 79476 | 3834 | 48.24 | 0.85 (0.75-0.96) |  |
|  | 150-300 | 8767 | 32942 | 1151 | 34.94 | 0.56 (0.49-0.64) |  |
|  | >300 | 3585 | 13545 | 381 | 28.13 | 0.41 (0.35-0.47) |  |
| Yes | 0 | 965 | 3280 | 555 | 169.21 |  |  |
|  | 0-150 | 868 | 3259 | 538 | 165.08 | 0.91 (0.77-1.07) |  |
|  | 150-300 | 399 | 1518 | 213 | 140.32 | 0.71 (0.58-0.88) |  |
|  | >300 | 134 | 541 | 66 | 122.00 | 0.79 (0.50-1.23) |  |

BMI, body mass index; SBP, systolic blood pressure; DBP, diastolic blood pressure; CCB, calcium-channel blocker; ACEI, angiotensin-converting enzyme inhibitor; ARB, angiotensin II receptor blocker; HR, hazard ratio, LTPA, leisure time physical activity.

| Table S5 Multivariable associations between CBD and time-updated LTPA estimated by the MSM within subgroups | | | | | | | |
| --- | --- | --- | --- | --- | --- | --- | --- |
| Characteristics | PAT | N | Person-years | Event | Event rates (1000PYs) | HR (95%CI) | P for Interaction |
|  |  |  |  |  |  |  |  |
| Age group |  |  |  |  |  |  | 0.573 |
| <60 | 0 | 10789 | 40129 | 1847 | 46.03 |  |  |
|  | 0-150 | 8837 | 35503 | 1350 | 38.02 | 0.86 (0.75-1.00) |  |
|  | 150-300 | 3797 | 14724 | 467 | 31.72 | 0.70 (0.59-0.84) |  |
|  | >300 | 1181 | 4855 | 124 | 25.54 | 0.36 (0.28-0.46) |  |
| 60-70 | 0 | 8385 | 28954 | 1964 | 67.83 |  |  |
|  | 0-150 | 7581 | 28979 | 1689 | 58.28 | 0.70 (0.60-0.82) |  |
|  | 150-300 | 3392 | 12366 | 525 | 42.46 | 0.44 (0.37-0.52) |  |
|  | >300 | 1723 | 6383 | 149 | 23.34 | 0.28 (0.22-0.34) |  |
| >70 | 0 | 4994 | 18094 | 1338 | 73.95 |  |  |
|  | 0-150 | 4696 | 18982 | 1140 | 60.06 | 0.80 (0.70-0.92) |  |
|  | 150-300 | 1977 | 7309 | 352 | 48.16 | 0.55 (0.46-0.64) |  |
|  | >300 | 815 | 2915 | 76 | 26.07 | 0.34 (0.26-0.43) |  |
| Gender |  |  |  |  |  |  | 0.526 |
| Women | 0 | 12989 | 47446 | 2989 | 63.00 |  |  |
|  | 0-150 | 11407 | 46056 | 2448 | 53.15 | 0.76 (0.70-0.82) |  |
|  | 150-300 | 4909 | 18472 | 726 | 39.30 | 0.52 (0.47-0.58) |  |
|  | >300 | 1895 | 7331 | 184 | 25.10 | 0.31 (0.26-0.37) |  |
| Men | 0 | 11179 | 39731 | 2160 | 54.37 |  |  |
|  | 0-150 | 9707 | 37408 | 1731 | 46.27 | 0.79 (0.64-0.97) |  |
|  | 150-300 | 4257 | 15928 | 618 | 38.80 | 0.56 (0.44-0.70) |  |
|  | >300 | 1824 | 6822 | 165 | 24.19 | 0.33 (0.26-0.44) |  |
| Medication |  |  |  |  |  |  | 0.467 |
| No | 0 | 10934 | 38538 | 2000 | 51.90 |  |  |
|  | 0-150 | 9766 | 35974 | 1648 | 45.81 | 0.79 (0.72-0.88) |  |
|  | 150-300 | 4071 | 14523 | 494 | 34.02 | 0.54 (0.48-0.61) |  |
|  | >300 | 1378 | 5161 | 102 | 19.76 | 0.35 (0.29-0.44) |  |
| Yes | 0 | 13234 | 48639 | 3149 | 64.74 |  |  |
|  | 0-150 | 11348 | 47490 | 2531 | 53.30 | 0.78 (0.68-0.90) |  |
|  | 150-300 | 5095 | 19877 | 850 | 42.76 | 0.56 (0.47-0.65) |  |
|  | >300 | 2341 | 8992 | 247 | 27.47 | 0.32 (0.26-0.38) |  |
| Diabetes mellitus | |  |  |  |  |  | 0.774 |
| No | 0 | 20798 | 76257 | 4438 | 58.20 |  |  |
|  | 0-150 | 18027 | 73018 | 3568 | 48.86 | 0.80 (0.73-0.88) |  |
|  | 150-300 | 7537 | 29134 | 1115 | 38.27 | 0.55 (0.50-0.61) |  |
|  | >300 | 3074 | 12084 | 287 | 23.75 | 0.33 (0.29-0.38) |  |
| Yes | 0 | 3370 | 10920 | 711 | 65.11 |  |  |
|  | 0-150 | 3087 | 10446 | 611 | 58.49 | 0.74 (0.63-0.87) |  |
|  | 150-300 | 1629 | 5266 | 229 | 43.49 | 0.53 (0.44-0.65) |  |
|  | >300 | 645 | 2070 | 62 | 29.95 | 0.33 (0.24-0.44) |  |
| Smoker |  |  |  |  |  |  | 0.003 |
| No | 0 | 19538 | 70759 | 4146 | 58.59 |  |  |
|  | 0-150 | 17083 | 68193 | 3382 | 49.59 | 0.62 (0.44-0.85) |  |
|  | 150-300 | 7422 | 27911 | 1030 | 36.90 | 0.41 (0.30-0.58) |  |
|  | >300 | 3135 | 11870 | 281 | 23.67 | 0.26 (0.18-0.36) |  |
| Yes | 0 | 4630 | 16418 | 1003 | 61.09 |  |  |
|  | 0-150 | 4031 | 15272 | 797 | 52.19 | 0.93 (0.80-1.08) |  |
|  | 150-300 | 1744 | 6489 | 314 | 48.39 | 0.74 (0.62-0.88) |  |
|  | >300 | 584 | 2283 | 68 | 29.79 | 0.43 (0.32-0.58) |  |
| Drinker |  |  |  |  |  |  | 0.001 |
| No | 0 | 20792 | 75121 | 4490 | 59.77 |  |  |
|  | 0-150 | 17741 | 70314 | 3537 | 50.30 | 0.73 (0.65-0.81) |  |
|  | 150-300 | 7704 | 28706 | 1110 | 38.67 | 0.50 (0.44-0.56) |  |
|  | >300 | 3119 | 11701 | 283 | 24.19 | 0.30 (0.26-0.35) |  |
| Yes | 0 | 3376 | 12056 | 659 | 54.66 |  |  |
|  | 0-150 | 3373 | 13150 | 642 | 48.82 | 0.99 (0.85-1.16) |  |
|  | 150-300 | 1462 | 5694 | 234 | 41.10 | 0.74 (0.61-0.91) |  |
|  | >300 | 600 | 2452 | 66 | 26.92 | 0.43 (0.32-0.59) |  |
| BMI |  |  |  |  |  |  | 0.371 |
| <24 | 0 | 8336 | 29254 | 1938 | 66.25 |  |  |
|  | 0-150 | 7534 | 29530 | 1616 | 54.72 | 0.77 (0.67-0.90) |  |
|  | 150-300 | 2816 | 10512 | 434 | 41.29 | 0.53 (0.44-0.62) |  |
|  | >300 | 962 | 3592 | 107 | 29.79 | 0.34 (0.27-0.43) |  |
| 24~27 | 0 | 9387 | 34085 | 2026 | 59.44 |  |  |
|  | 0-150 | 8340 | 33103 | 1612 | 48.70 | 0.91 (0.79-1.04) |  |
|  | 150-300 | 3751 | 14245 | 585 | 41.07 | 0.67 (0.56-0.80) |  |
|  | >300 | 1451 | 5567 | 141 | 25.33 | 0.40 (0.32-0.51) |  |
| >27 | 0 | 6445 | 23839 | 1185 | 49.71 |  |  |
|  | 0-150 | 5240 | 20831 | 951 | 45.65 | 0.73 (0.63-0.85) |  |
|  | 150-300 | 2599 | 9643 | 325 | 33.70 | 0.50 (0.42-0.59) |  |
|  | >300 | 1306 | 4994 | 101 | 20.22 | 0.29 (0.23-0.37) |  |
| Hyperlipidemia | |  |  |  |  |  | 0.073 |
| No | 0 | 23203 | 83818 | 4620 | 55.12 |  |  |
|  | 0-150 | 20246 | 80041 | 3727 | 46.56 | 0.76 (0.69-0.84) |  |
|  | 150-300 | 8767 | 32801 | 1168 | 35.61 | 0.53 (0.47-0.59) |  |
|  | >300 | 3585 | 13595 | 305 | 22.43 | 0.31 (0.27-0.36) |  |
| Yes | 0 | 965 | 3359 | 529 | 157.49 |  |  |
|  | 0-150 | 868 | 3423 | 452 | 132.05 | 0.82 (0.69-0.98) |  |
|  | 150-300 | 399 | 1599 | 176 | 110.07 | 0.62 (0.50-0.77) |  |
|  | >300 | 134 | 559 | 44 | 78.71 | 0.38 (0.25-0.57) |  |

BMI, body mass index; SBP, systolic blood pressure; DBP, diastolic blood pressure; CCB, calcium-channel blocker; ACEI, angiotensin-converting enzyme inhibitor; ARB, angiotensin II receptor blocker; HR, hazard ratio, LTPA, leisure time physical activity.

| Table S6 Multivariable associations between CVD and LTPA estimated by the MSM within subgroups | | | | | | |
| --- | --- | --- | --- | --- | --- | --- |
| Characteristics | N | Person-years | Events | Event rates (1000PYs) | HR (95%CI) | *P* for Interaction |
|  |  |  |  |  |  |  |
| Age group | |  |  |  |  | 0.753 |
| <60 | 24604 | 91494 | 5644 | 61.69 | 0.85 (0.81-0.88) |  |
| 60-70 | 21081 | 71814 | 6444 | 89.73 | 0.87 (0.83-0.91) |  |
| >70 | 12482 | 43851 | 4244 | 96.78 | 0.86 (0.85-0.88) |  |
| Gender |  |  |  |  |  | 0.114 |
| Women | 31200 | 112261 | 9361 | 83.39 | 0.92 (0.84-1.02) |  |
| Men | 26967 | 94898 | 6971 | 73.46 | 0.86 (0.84-0.89) |  |
| Diabetes mellitus | |  |  |  |  | 0.276 |
| No | 49436 | 180272 | 13909 | 77.16 | 0.84 (0.8-0.89) |  |
| Yes | 8731 | 26888 | 2423 | 90.11 | 0.89 (0.87-0.92) |  |
| Smoker |  |  |  |  |  | 0.011 |
| No | 47178 | 169021 | 13132 | 77.69 | 0.85 (0.81-0.89) |  |
| Yes | 10989 | 38138 | 3200 | 83.91 | 0.88 (0.86-0.9) |  |
| Drinker |  |  |  |  |  | 0.531 |
| No | 49356 | 175539 | 13976 | 79.62 | 0.85 (0.81-0.89) |  |
| Yes | 8811 | 31620 | 2356 | 74.51 | 0.88 (0.85-0.9) |  |
| BMI |  |  |  |  |  | 0.017 |
| <24 | 19648 | 68374 | 5910 | 86.44 | 0.81 (0.77-0.86) |  |
| 24~27 | 22929 | 81841 | 6415 | 78.38 | 0.9 (0.85-0.95) |  |
| >27 | 15590 | 56944 | 4007 | 70.37 | 0.88 (0.85-0.9) |  |
| Medication | |  |  |  |  | 0.112 |
| No | 26149 | 89714 | 6288 | 70.09 | 0.93 (0.85-1.02) |  |
| Yes | 32018 | 117445 | 10044 | 85.52 | 0.85 (0.82-0.88) |  |
| Hyperlipidemia | |  |  |  |  | <0.001 |
| No | 55801 | 199353 | 14661 | 73.54 | 0.85 (0.82-0.88) |  |
| Yes | 2366 | 7807 | 1671 | 214.04 | 0.91 (0.88-0.94) |  |

BMI, body mass index; SBP, systolic blood pressure; DBP, diastolic blood pressure; CCB, calcium-channel blocker; ACEI, angiotensin-converting enzyme inhibitor; ARB, angiotensin II receptor blocker; HR, hazard ratio, LTPA, leisure time physical activity.

| Table S7 Multivariable associations between CHD and LTPA estimated by the MSM within subgroups | | | | | | |
| --- | --- | --- | --- | --- | --- | --- |
| Characteristics | N | Person-years | Events | Event rates (1000PYs) | HR (95%CI) | *P* for Interaction |
|  |  |  |  |  |  |  |
| Age group | |  |  |  |  | 0.753 |
| <60 | 24604 | 95955 | 3551 | 37.01 | 0.86 (0.82-0.91) | |
| 60-70 | 21081 | 76452 | 4458 | 58.31 | 0.88 (0.84-0.93) | |
| >70 | 12482 | 46623 | 2996 | 64.26 | 0.88 (0.86-0.90) | |
| Gender |  |  |  |  |  | 0.114 |
| Women | 31200 | 119068 | 6357 | 53.39 | 0.95 (0.85-1.06) | |
| Men | 26967 | 99961 | 4648 | 46.50 | 0.87 (0.84-0.90) | |
| Diabetes mellitus | |  |  |  |  | 0.276 |
| No | 49436 | 190545 | 9320 | 48.91 | 0.85 (0.81-0.90) | |
| Yes | 8731 | 28485 | 1685 | 59.15 | 0.92 (0.89-0.95) | |
| Smoker |  |  |  |  |  | 0.011 |
| No | 47178 | 178412 | 8952 | 50.18 | 0.86 (0.82-0.91) | |
| Yes | 10989 | 40618 | 2053 | 50.54 | 0.89 (0.87-0.92) | |
| Drinker |  |  |  |  |  | 0.531 |
| No | 49356 | 185642 | 9490 | 51.12 | 0.86 (0.82-0.91) | |
| Yes | 8811 | 33388 | 1515 | 45.38 | 0.89 (0.86-0.92) | |
| BMI |  |  |  |  |  | 0.017 |
| <24 | 19648 | 72739 | 3968 | 54.55 | 0.82 (0.77-0.87) | |
| 24~27 | 22929 | 86588 | 4312 | 49.80 | 0.93 (0.87-0.99) | |
| >27 | 15590 | 59703 | 2725 | 45.64 | 0.89 (0.86-0.93) | |
| Medication | |  |  |  |  | 0.112 |
| No | 26149 | 94405 | 4100 | 43.43 | 0.96 (0.86-1.06) | |
| Yes | 32018 | 124625 | 6905 | 55.41 | 0.86 (0.83-0.89) | |
| Hyperlipidemia | |  |  |  |  | <0.001 |
| No | 55801 | 210432 | 9633 | 45.78 | 0.86 (0.82-0.89) | |
| Yes | 2366 | 8597 | 1372 | 159.59 | 0.95 (0.91-0.99) | |

BMI, body mass index; SBP, systolic blood pressure; DBP, diastolic blood pressure; HR, hazard ratio, LTPA, leisure time physical activity.

| Table S8 Multivariable associations between CBD and LTPA estimated by the MSM within subgroups | | | | | | |
| --- | --- | --- | --- | --- | --- | --- |
| Characteristics | N | Person-years | Events | Event rates (1000PYs) | HR (95%CI) | *P* for Interaction |
|  |  |  |  |  |  |  |
| Age group | |  |  |  |  | 0.753 |
| <60 | 24604 | 95212 | 3788 | 39.78 | 0.83 (0.80-0.87) |  |
| 60-70 | 21081 | 76682 | 4327 | 56.43 | 0.84 (0.81-0.87) |  |
| >70 | 12482 | 47300 | 2906 | 61.44 | 0.85 (0.83-0.88) |  |
| Gender |  |  |  |  |  | 0.114 |
| Women | 31200 | 119305 | 6347 | 53.20 | 0.82 (0.80-0.85) |  |
| Men | 26967 | 99889 | 4674 | 46.79 | 0.86 (0.83-0.88) |  |
| Diabetes mellitus | |  |  |  |  | 0.276 |
| No | 49436 | 190492 | 9408 | 49.39 | 0.83 (0.79-0.86) |  |
| Yes | 8731 | 28702 | 1613 | 56.20 | 0.86 (0.83-0.89) |  |
| Smoker |  |  |  |  |  | 0.011 |
| No | 47178 | 178733 | 8839 | 49.45 | 0.83 (0.79-0.86) |  |
| Yes | 10989 | 40461 | 2182 | 53.93 | 0.88 (0.85-0.90) |  |
| Drinker |  |  |  |  |  | 0.531 |
| No | 49356 | 185842 | 9420 | 50.69 | 0.83 (0.79-0.86) |  |
| Yes | 8811 | 33352 | 1601 | 48.00 | 0.87 (0.85-0.90) |  |
| BMI |  |  |  |  |  | 0.017 |
| <24 | 19648 | 72887 | 4095 | 56.18 | 0.82 (0.78-0.86) |  |
| 24~27 | 22929 | 87001 | 4364 | 50.16 | 0.85 (0.83-0.87) |  |
| >27 | 15590 | 59306 | 2562 | 43.20 | 0.86 (0.82-0.89) |  |
| Medication | |  |  |  |  | 0.112 |
| No | 26149 | 94196 | 4244 | 45.05 | 0.85 (0.83-0.87) |  |
| Yes | 32018 | 124998 | 6777 | 54.22 | 0.84 (0.81-0.86) |  |
| Hyperlipidemia | |  |  |  |  | <0.001 |
| No | 55801 | 210255 | 9820 | 46.71 | 0.83 (0.81-0.86) |  |
| Yes | 2366 | 8939 | 1201 | 134.36 | 0.84 (0.80-0.88) |  |

BMI, body mass index; SBP, systolic blood pressure; DBP, diastolic blood pressure; CCB, calcium-channel blocker; ACEI, angiotensin-converting enzyme inhibitor; ARB, angiotensin II receptor blocker; HR, hazard ratio, LTPA, leisure time physical activity.

| Table S9 the parameters of the curvilinear relationship between CVD events and LTPA | | | | |
| --- | --- | --- | --- | --- |
| LTPA | Slope | Stand error | 95%CI Lower | 95%CI Upper |
| CVD |  |  |  |  |
| LTPA≤417 min | -0.0017 | 0.0001 | -0.0018 | -0.0015 |
| LTPA>417 min | -0.0003 | 0.0005 | -0.0013 | 0.0008 |
| CHD |  |  |  |  |
| LTPA≤419 min | -0.0013 | 0.0001 | -0.0016 | -0.0011 |
| LTPA>419 min | -0.0005 | 0.0003 | -0.0012 | 0.0001 |
| CBD |  |  |  |  |
| LTPA≤413 min | -0.0020 | 0.0001 | -0.0022 | -0.0018 |
| LTPA>413 min | -0.0001 | 0.0007 | -0.0014 | 0.0012 |

CVD, cardiovascular disease; CHD, coronary heart diseases; CBD, cerebrovascular diseases; LTPA, leisure time physical activity;


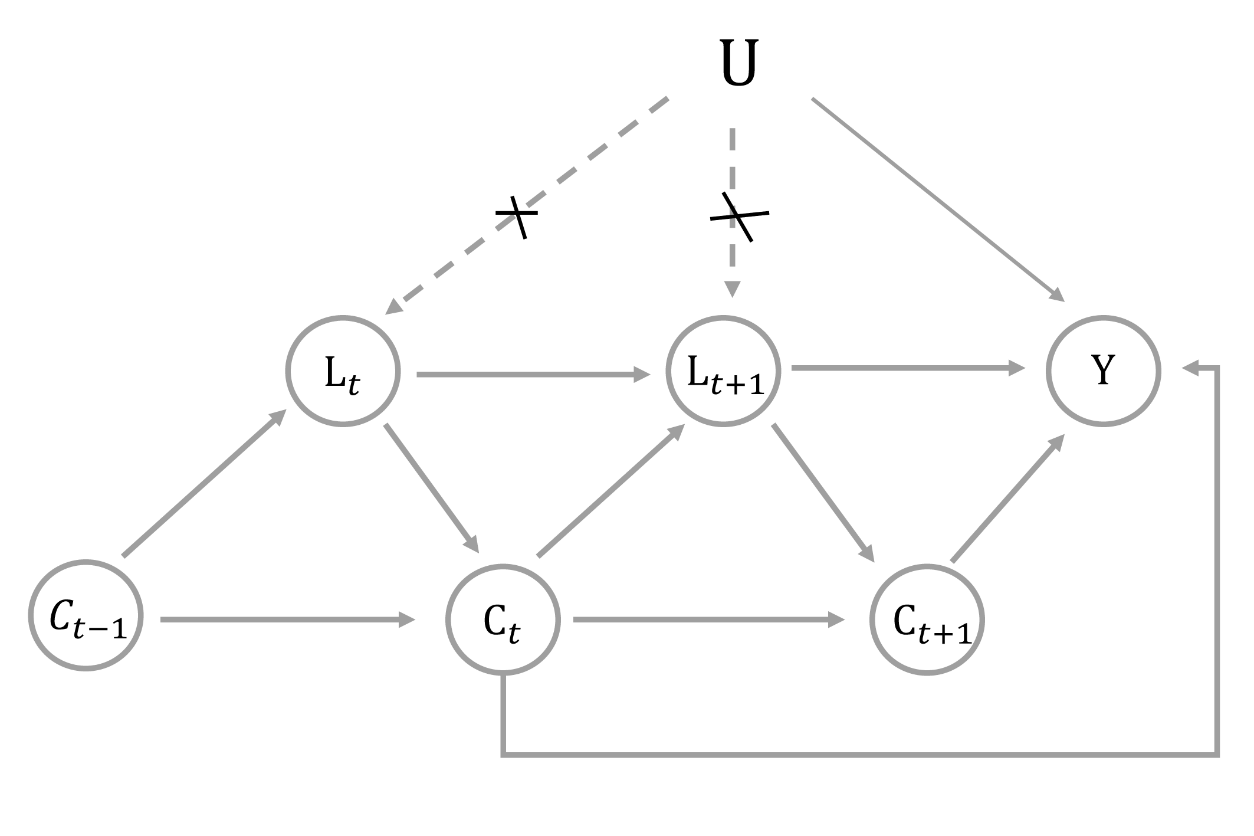


Figure S1. A diagram of time-dependent confounder.$C_{t}$, time-dependent confounder;$L_{t}$, measurement of LTPA at time *t*; U, unobservable factors; Y, the outcomes; The MSMs are performed under the assumption of no unobserved confounders, that is, unconfoundedness assumption. LTPA, leisure time physical activity.


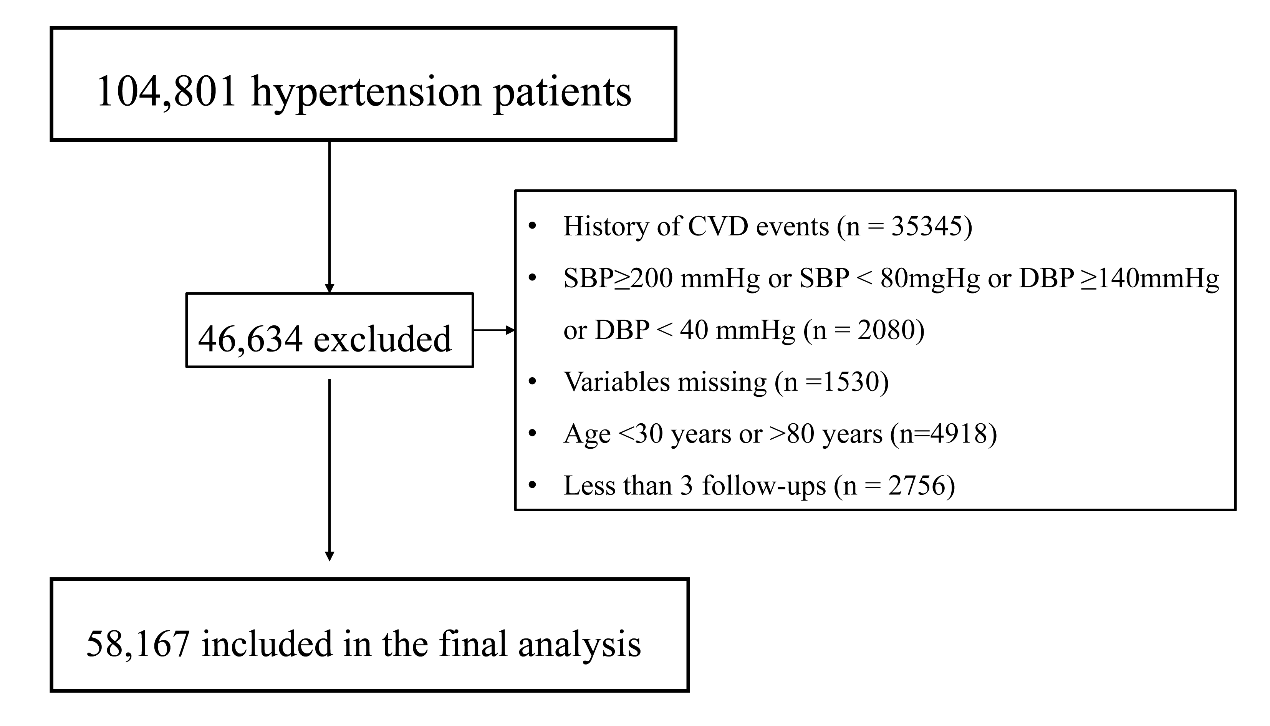


Figure S2. Flowchart showing numbers of patients excluded from the analysis. CVD, cardiovascular diseases; SBP, systolic blood pressure; DBP, diastolic blood pressure;


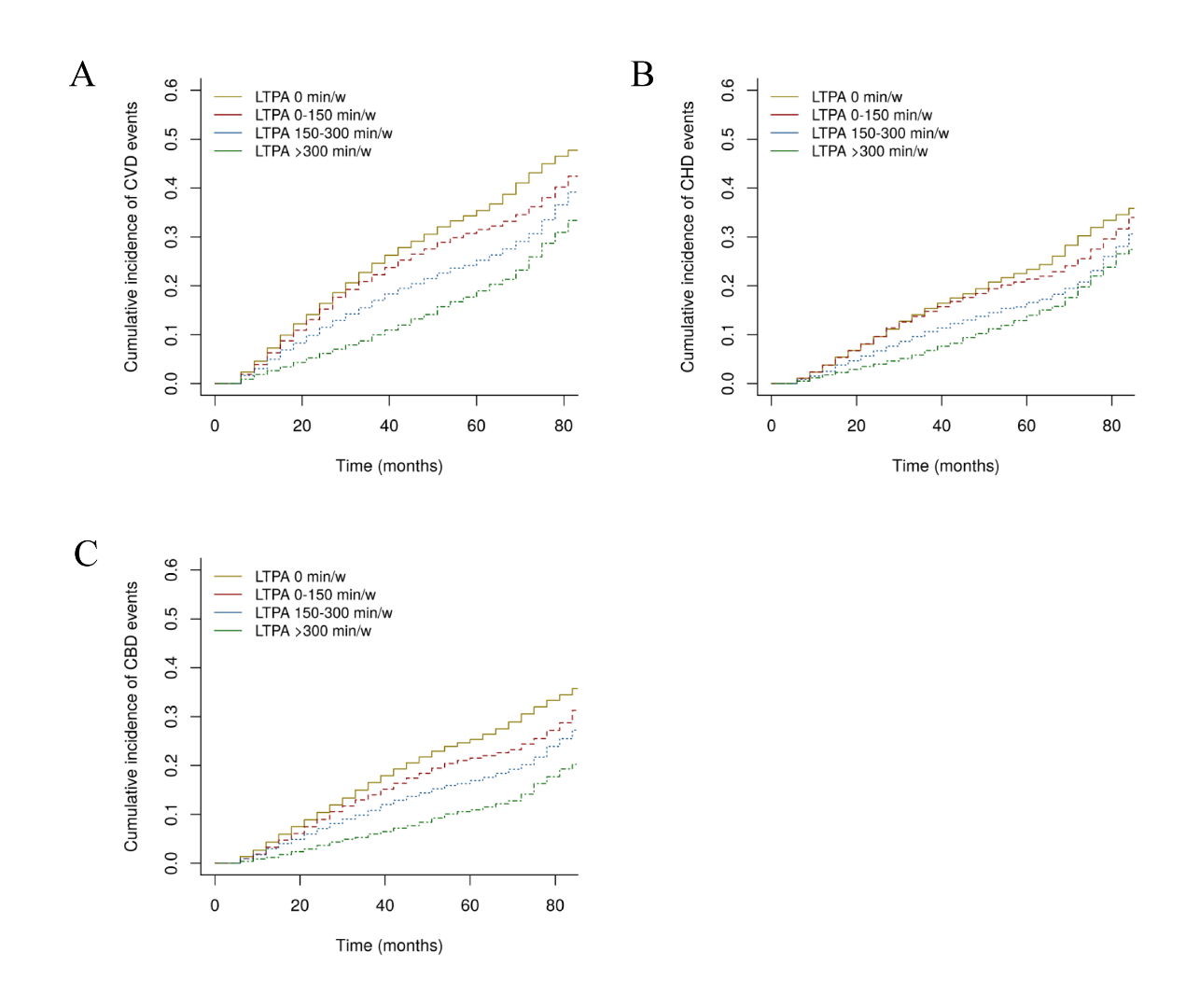


Figure S3. Cumulative incidence functions of CVD (A), CHD (B), CBD (C) across categories of LTPA at baseline among patients of this study. CVD, cardiovascular diseases; CHD, coronary heart disease; CBD, cerebrovascular diseases; LTPA, leisure time physical activity;
